# Perceived Stress and Subjective Happiness in Medical Students Before and After a Wellness Speaker Session

**DOI:** 10.7759/cureus.104477

**Published:** 2026-03-01

**Authors:** Smaran Marupudi, Jared Hensley, Sriya Gullapalli, David Sta Maria, Nikhilesh Anand

**Affiliations:** 1 Department of Medical Education, University of Texas Rio Grande Valley School of Medicine, Edinburg, USA

**Keywords:** medical school, medical student wellness, perceived stress scale, stress management, subjective happiness scale

## Abstract

Background: Medical students experience psychological stress due to academic workload, thereby leading to a reduction in overall well-being. In response, medical schools are incorporating wellness activities into the curriculum. However, single-session events have not been rigorously evaluated. This pilot study assessed the feasibility and acceptability of a single-session speaker event and examined pre- versus post-event differences in mean stress and happiness scores among survey respondents.

Methods: This study used anonymous, unlinked, pre- and post-event cross-sectional surveys to compare group-level perceived stress and subjective happiness among medical students attending a single-session wellness speaker event. First- and second-year medical students completed validated pre- and post-surveys, including the Perceived Stress Scale (PSS) and the Subjective Happiness Scale (SHS). The 60-min event, which was organized by student investigators, took place in January 2025. Welch’s independent t-tests were used to compare group means. Effect sizes were calculated using Cohen’s d. Post-event perception items were summarized descriptively, and open-ended responses were reviewed for recurring themes.

Results: A total of 76 pre-surveys and 55 post-surveys were collected from 110 first- and second-year medical students. No statistically significant differences were found across PSS or SHS items (p>0.05), and effect sizes were uniformly small (Cohen’s d range: -0.25 to +0.26). Despite the absence of statistically significant quantitative differences, 46/55 (83.6%) students reported that the event was helpful, 31/55 (57%) felt more confident managing stress, and the mean satisfaction score was 7.4/10. Open-ended feedback highlighted the speaker’s relatability. Views were mixed on curricular integration, with 28/55 (50.9%) in favor of including similar wellness events as part of medical school programming.

Conclusion: This pilot study supports the feasibility and acceptability of brief, speaker-based wellness interventions in medical education. While no statistically significant group-level differences were detected in the immediate post-event analysis, student feedback suggests perceived value. Future efforts should explore longitudinal formats with supplemental training events and linked pre- and post-measurement to better assess outcomes and inform curriculum development.

## Introduction

Medical students report elevated levels of psychological distress compared to the general population, with high frequencies of depression (27-32%) [1-3], anxiety (20-33%) [1,4,5], and stress (41%) [1]. A variety of factors are involved, including workload, financial concerns, academic performance, and sleep deprivation, among others [6-8]. These challenges can negatively impact learning, empathy, long-term professional development, and overall health [8], with 22% of U.S. medical students having taken or considered taking a leave of absence for personal well-being [9]. In response, medical schools have implemented longitudinal wellness initiatives. In one national survey of 27 U.S. medical schools, 16 (59%) reported having a formal student well-being curriculum, with 13 (81%) of these programs having content integrated into regular curricular hours [10]. One example includes the Practice Enhancement, Engagement, Resilience, and Support (PEERS) program at Mount Sinai. This program provides 10 small-group sessions that emphasize mindfulness, positive psychology, and principles of cognitive and dialectical-behavioral therapy throughout the four years of medical school [11]. Another is the Recognize, Empathize, Allow, Care, Hold each other up (REACH) curriculum at the Medical College of Wisconsin, which uses trauma stewardship principles to prepare students for the emotional challenges of medicine [12].

Interestingly, the emerging literature suggests that even short-term, informal interventions are associated with measurable benefits in perceived stress among medical students. For example, the results of one meta-analysis suggest that shorter interventions with fewer required practice hours may improve stress [13]. The results of another study among Australian medical students found that improvements in mental health, perceived stress, study engagement, and mindfulness were positively correlated with informal practice [14]. Despite this, few studies have evaluated the feasibility, acceptability, and pre- versus post-differences associated with single-session interventions designed for early implementation in medical education.

To address the need for scalable, low-effort wellness programming, we developed and piloted Prescribing Happiness, a one-time, 60-min wellness speaker session offered during a high-stress academic period for first- and second-year medical students. The session integrated storytelling, evidence-based strategies, and reflective exercises to address themes of happiness, stress management, and emotional resilience. Rather than being delivered during a protected or ideal time, such as orientation, this intervention was implemented during a period of active coursework, allowing us to assess its feasibility under real-world academic conditions. Primary outcomes included measurement of feasibility and acceptability metrics. Feasibility was operationalized as the ability to implement a low-resource 60-min wellness session within the existing curriculum and to obtain voluntary pre- and post-event responses. Acceptability metrics included survey response rates along with post-event feedback. Secondary outcomes included pre- and post-event differences in perceived stress and subjective happiness scores. We hypothesized that post-event respondents would report lower perceived stress and higher subjective happiness compared with pre-event respondents.

## Materials and methods

The study protocol was approved by the University of Texas Rio Grande Valley (UTRGV) Institutional Review Board (#IRB-25-0011). This study employed an unlinked pre-post survey design to assess group-level differences in pre- and post-event responses among medical students attending a wellness speaker intervention. The pre-survey consisted of demographics (year in medical school and age category) in addition to questions from both scales. The post-survey consisted of both scales in addition to post-event feedback questions. Both instruments can be found in the appendix section. Table 1 presents the post-event feedback questions and answer choices.

**Table 1:** Post-event feedback questions and response rates (n=55).

Questions	Answer choices and results
Did you find the wellness speaker event helpful?	Yes: 46/55 (83.6%), no: 9/55 (16.4%)
To what extent do you feel the speaker's message was relevant to the challenges of medical school?	Strongly disagree: 2/55 (3.6%), disagree: 4/55 (7.3%), neutral: 11/55 (20.0%), agree: 19/55 (34.5%), strongly agree: 19/55 (34.5%)
Do you feel more confident in your ability to manage stress and maintain well-being after attending this event?	Strongly disagree: 4/55 (7.3%), disagree: 4/55 (7.3%), neutral: 16/55 (29.1%), agree: 26/55 (47.3%), strongly agree: 5/55 (9.1%)
Would you like to see similar wellness-focused events as part of the medical school curriculum?	Yes: 28/55 (50.9%), no: 27/55 (49.1%)
Do you think integrating wellness events like this one could help improve medical students' mental health over time?	Strongly disagree: 4/55 (7.3%), disagree: 6/55 (10.9%), neutral: 10/55 (18.2%), agree: 24/55 (43.6%), strongly agree: 11/55 (20.0%)
On a scale of 1-10, how satisfied were you with the speaker and the content of the session?	Slidable scale: not at all satisfied (1) -> extremely satisfied (10); minimum: 1; mean: 7.4; maximum: 10
Any feedback that you would like to provide for this event?	Free-response; themes summarized in qualitative feedback section

The intervention took place on January 29, 2025, in the University of Texas Rio Grande Valley (UTRGV) School of Medicine auditorium and consisted of a 60-min presentation delivered by an invited physician speaker with lived experience related to physician wellness. The interactive session utilized multi-modal media, including short videos and excerpts from research studies, to engage participants. It had the following chronological structure: (1) a personal narrative component, (2) discussion of evidence-informed stress management techniques, and (3) audience reflection and question-and-answer discussion. The session integrated discussion of experiences within and outside of medical training with broader themes of gratitude, stress management, and strategies for maintaining personal well-being. To protect speaker confidentiality, the speaker’s identity and personal identifying details are not disclosed in this manuscript.

Participants

A total of 110 first- and second-year medical students attended the mandatory event and were invited to participate in an optional survey. A total of 76 students completed the pre-survey (32 first-year, 44 second-year), and 55 completed the post-survey (24 first-year, 31 second-year). Participation in the surveys was voluntary and anonymous. No incentives were provided.

Survey architecture

Students provided their classification in medical school, along with their age (categorized). To assess perceived stress, students completed the 10-item Perceived Stress Scale (PSS-10), a widely validated instrument developed by Cohen et al. in 1983 [15,16]. The 10-item version was derived from the original 14-item Perceived Stress Scale by selecting items with the strongest psychometric properties [16]. Each question is scored on a five-point Likert scale ranging from 0 (never) to 4 (very often), generating total scores between 0 and 40, with higher scores indicating greater perceived stress [15,17]. It is one of the three most common tools used for measuring psychological distress in medical students, appearing in 14.7% of studies [18], demonstrating high internal consistency with strong reliability and validity across gender, age, and clinical status [19-22].

To assess well-being more comprehensively beyond perceived stress alone, students completed the four-item Subjective Happiness Scale (SHS), which evaluates global happiness and life satisfaction as independent factors [23-25]. The scale was originally developed by Lyubomirsky and Lepper in 1999 [25]. Each item is rated on a seven-point scale for a total score between 4 and 28, where higher scores indicate a greater degree of subjective happiness [25]. Similar to PSS-10, it demonstrates strong internal validity and has been utilized to assess medical student populations [23,25-27]. Additional items included post-event feedback questions developed by the study team based on prior wellness programming evaluations and revised for clarity prior to administration. These questions assessed student satisfaction, perceived relevance, confidence in managing stress, attitudes toward curricular integration of wellness programming, and qualitative feedback (Table 1).

Procedure

Informed consent was obtained from students prior to completing the survey. Each form was electronically administered using REDCap software (Nashville, Tennessee: Vanderbilt University Medical Center) during the evening prior to the event. Students who did not complete the survey that evening were asked to complete the survey prior to the wellness session. The post-survey was administered immediately after the event, and responses were collected for the subsequent 24 h. Due to the anonymous design, individual responses could not be linked across timepoints.

Data analysis

All data were analyzed using Microsoft Excel version 16.77.1 (Redmond, WA: Microsoft Corp.). Because pre- and post-cross-sectional surveys were anonymous and unlinked, comparisons reflect independent sample differences rather than within-individual change. Given sample sizes at each time point, mean estimates were assumed to be approximately normally distributed under the central limit theorem. Welch’s independent t-tests were used to compare group means for each survey item, accounting for unequal sample sizes and variances. Means were calculated using only available responses (available-case analysis). Given multiple item-level comparisons, findings were interpreted cautiously due to the increased risk of type I error. Cohen’s d was calculated to estimate the effect size. P<0.05 was considered statistically significant. Post-survey perception items were summarized using descriptive statistics. Two members of the study team independently reviewed responses and identified recurrent themes through inductive thematic analysis, with discrepancies resolved through discussion.

## Results

A total of 76 first- and second-year medical students completed the pre-event survey, and 55 students completed the post-event survey. Due to the anonymous survey design, individual responses could not be linked across time points.

Perceived Stress Scale (PSS)

The PSS-10 assessed stress using a five-point Likert scale (0=never, 4=very often). Across the 10 items, no statistically significant differences in mean perceived stress item-scores were observed between pre- and post-event responses.

Subjective Happiness Scale (SHS)

The SHS used a seven-point Likert scale (1=not at all happy, 7=very happy). Across all four SHS items, no statistically significant differences in subjective happiness item scores were detected. Full item-level statistics for both scales are reported in Tables 2, 3.

**Table 2:** PSS-10 results (Welch's independent t-test). Responses were graded on a five-point Likert scale. Participants selected from the following answer choices: 0=never, 1=almost never, 2=sometimes, 3=fairly often, and 4=very often. PSS-10: 10-item Perceived Stress Scale

Perceived Stress Scale: in the last month, how often have you…	Pre-mean (n=76)	Post-mean (n=55)	Pre-post mean difference	Test-statistic	p-Value (α=0.05)	Effect size (Cohen's d)
…been upset because of something that happened unexpectedly?	3.24	3.09	0.15	0.82	0.42	0.14
…felt that you were unable to control the important things in your life?	2.87	2.85	0.01	0.07	0.94	0.01
…felt nervous and “stressed”?	3.79	3.69	0.10	0.53	0.60	0.09
…felt confident about your ability to handle your personal problems?	3.92	3.71	0.21	1.35	0.18	0.24
…felt things were going your way?	3.50	3.33	0.17	1.15	0.25	0.21
…found that you could not cope with all the things that you had to do?	2.66	2.93	-0.27	1.42	0.16	-0.25
…been able to control irritations in your life?	3.57	3.35	0.22	1.52	0.13	0.26
…felt that you were on top of things?	3.16	3.16	-0.01	0.03	0.97	-0.01
…been angered because of things that happened that were outside of your control?	2.92	2.95	-0.02	0.13	0.89	-0.02
…felt difficulties were piling up so high that you could not overcome them?	2.87	2.96	-0.10	0.45	0.65	-0.08

**Table 3:** SHS results (Welch's independent t-test). Responses were graded on a seven-point scale. Responses to question 1 ranged from “not a very happy person” to “a very happy person.” Responses to question 2 ranged from “less happy” to “more happy.” Responses to questions three and four ranged from “not at all” to “a great deal.” SHS: Subjective Happiness Scale

Subjective Happiness Scale (SHS)	Pre-mean (n=76)	Post-mean (n=55)	Pre-post mean difference	Test-statistic	p-Value (α=0.05)	Effect size (Cohen's d)
1. In general, I consider myself: not a very happy person -> a very happy person	5.11	5.04	0.07	0.31	0.757	0.06
2. Compared to most of my peers, I consider myself: less happy -> more happy	4.61	4.71	-0.1	0.41	0.682	-0.07
3. Some people are generally very happy. They enjoy life regardless of what is going on, getting the most out of everything. To what extent does this characterization describe you? Not at all -> a great deal	4.38	4.62	-0.24	0.96	0.337	-0.17
4. Some people are generally not very happy. Although they are not depressed, they never seem as happy as they might be. To what extent does this characterization describe you? Not at all -> a great deal	3.49	3.55	-0.06	0.21	0.838	-0.04

Post-survey perceptions

Despite no significant differences between samples, participants reported high satisfaction. Eighty-four percent (46/55) of students found the event helpful, and 57% (31/55) felt more confident in managing stress. The mean satisfaction rating was 7.4 out of 10, and 28/55 (51%) supported integrating similar events into the curriculum, while 27/55 (49%) preferred non-mandatory formats. Questions and associated response rates are reported in Table 1.

Qualitative feedback

Open-ended responses were reviewed and grouped into common themes. The analysis revealed themes of emotional validation and speaker relatability, along with constructive suggestions for improvement. Participants generally expressed appreciation for the speaker’s message and described the session as a meaningful reminder to prioritize gratitude and mental well-being during medical training. In contrast, several responses highlighted logistical concerns, particularly regarding mandatory attendance and scheduling proximal to examinations, which were perceived as stress-inducing. Additional feedback suggested that future sessions may benefit from increased tailoring of content to the specific experiences and challenges of medical students.

Attrition analysis

Of the 76 pre-event respondents, 55 completed the post-event survey (72% retention). We conducted an attrition analysis utilizing two demographic variables (year in training and age category) to examine variations between the two groups. Year in training (first- or second-year medical student) did not differ significantly between pre- and post-event respondents (χ²=0.03, p=0.86; pre n=76, post n=55). Age distribution was similarly comparable (χ²=0.24, p=0.97; pre n=73, post n=55), suggesting no evidence of demographic differences across time points. While demographic characteristics were similar across time points, we were unable to assess whether groups varied based on unmeasured factors such as baseline stress level or attitudes toward wellness programming.

## Discussion

This pilot study evaluated feasibility and immediate pre- versus post-event differences in stress and happiness scores among medical students attending a single-session wellness speaker event. While no statistically significant differences were observed in either the Perceived Stress Scale (PSS) or Subjective Happiness Scale (SHS), the event was implemented and positively received with voluntary survey participation, highlighting the feasibility and acceptability of brief, narrative-driven wellness interventions in medical education [1,9].

Our findings align with existing literature suggesting that short-term wellness interventions, particularly one-time sessions, often yield limited measurable changes in psychological outcomes when assessed immediately afterward [4,11,12]. Similar studies have found that while quantitative improvements may be small or non-significant, subjective perceptions of benefit remain high, especially when interventions are perceived as relatable or emotionally validating [9,12]. For example, a study of a single reflective writing workshop among obstetrics and gynecology residents showed minimal change in burnout scores but high ratings of personal insight and peer connection [28]. This reflects a broader challenge in wellness research as follows: standard scales like PSS or SHS may not capture the nuanced, short-term emotional shifts or cultural effects of wellness programming [5,9]. Furthermore, data from Canadian medical students demonstrates that medical students aged 20-34 years have significantly higher rates of diagnosed mood disorders, anxiety disorders, suicidal ideation, and suicidal distress compared to age-matched graduate students [29]. Eighty-nine percent of our participants were between the ages of 22 and 30 years, highlighting the importance of such an intervention.

In this study, the speaker’s personal story and authenticity were frequently highlighted in qualitative feedback, suggesting that narrative resonance may be a key mechanism underlying perceived benefit. Prior literature supports the role of storytelling and vulnerability in fostering a more compassionate learning environment and reducing stigma around mental health help-seeking [9]. For instance, the previously discussed Recognize, Empathize, Allow, Care, Hold each other up (REACH) curriculum incorporated the sharing of personal stories by instructors during didactics and small-group sessions. This program has been received positively by students, with the majority reporting improvements in self-care, mindfulness, and aid-seeking skills [12]. These narrative-driven models may serve as bridges toward more structured or skills-based wellness programming [8,12].

Despite the minimal quantitative differences, post-event feedback showed that more than 80% of participants reported that the event was helpful, and over half expressed increased confidence in managing stress. These findings suggest the event was perceived as being supportive of students' self-efficacy, an outcome that may require repeated or skill-based reinforcement to produce lasting behavioral change [11]. Qualitative feedback further suggests that logistical factors, such as timing and perceived autonomy of participation, may influence student engagement and subjective responses to wellness initiatives, independent of content quality.

Future directions

Building on these findings, future wellness initiatives may consider incorporating multi-session interventions with study populations expanded to third- and fourth-year medical students. The addition of supplementary “implementation” sessions focused on mindfulness, time management, or coping strategy workshops may complement speaker events and promote active learning [4,11]. Furthermore, linking pre- and post-survey responses at the individual level will enable paired statistical analysis and better detect meaningful within-subject changes [6]. Finally, future studies should use mixed methods approaches, including follow-up interviews or focus groups, to capture emotional impact, identity shifts, or perceived cultural changes not easily measured by quantitative scales [7,8].

Limitations

This study has several limitations. The modest sample size, lack of a control group, and no linked pre- and post-survey data prevented assessment of within-individual change and precluded causal inference. Attrition between the pre- and post-surveys (76-55 respondents) raises the possibility of selection bias, as differences between respondents at each time point could not be determined. The reduced post-survey sample size also limited the ability to detect the small effect sizes, increasing the risk of a type II error. Additionally, data analysis involved multiple item-level comparisons without adjustment, increasing the risk of type I error.

Furthermore, the Perceived Stress Scale assesses changes over the past month, which may not be adequately sensitive to 1-h changes following a single-hour intervention. Outcomes were assessed immediately following the session, with no longitudinal follow-up, preventing analysis of the durability of observed differences. Post-event perception responses may also reflect potential social desirability bias, as participants may desire to portray a more socially favorable response to the event. Likewise, the mandatory nature of this speaker session may also have affected students' perception and engagement. Overall, generalizability is limited due to the single-session, single-cohort, and single-institution design. Despite these limitations, this pilot study contributes to the growing literature on wellness programming in medical education and provides a foundation for future research and program development.

## Conclusions

Although this single-session wellness speaker event did not show statistically significant differences in perceived stress or happiness, it was successfully implemented and positively received by participating medical students. The majority of students found the event helpful, and over half reported feeling more confident in their ability to manage stress. These findings suggest that brief, narrative-driven interventions are feasible and offer perceived value and emotional support within the context of medical student education.

Future wellness programming should consider incorporating multi-session or longitudinal formats, linking pre- and post-survey data, and exploring a variety of supplemental delivery models to better address diverse student preferences. Institutions may benefit from treating wellness not solely as an outcome, but as a process of ongoing engagement, reflection, and cultural change. This study adds to the growing evidence base evaluating the role of structured, student-centered wellness interventions in medical education.

## Figures and Tables

**Figure 1:**
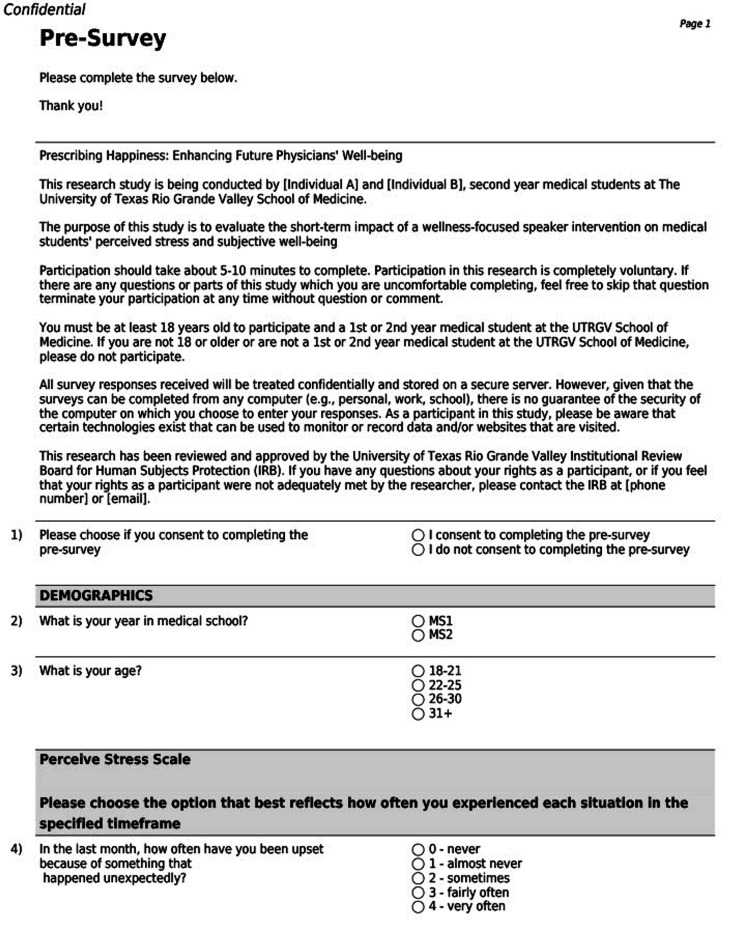
Pre-survey (page 1).

**Figure 2:**
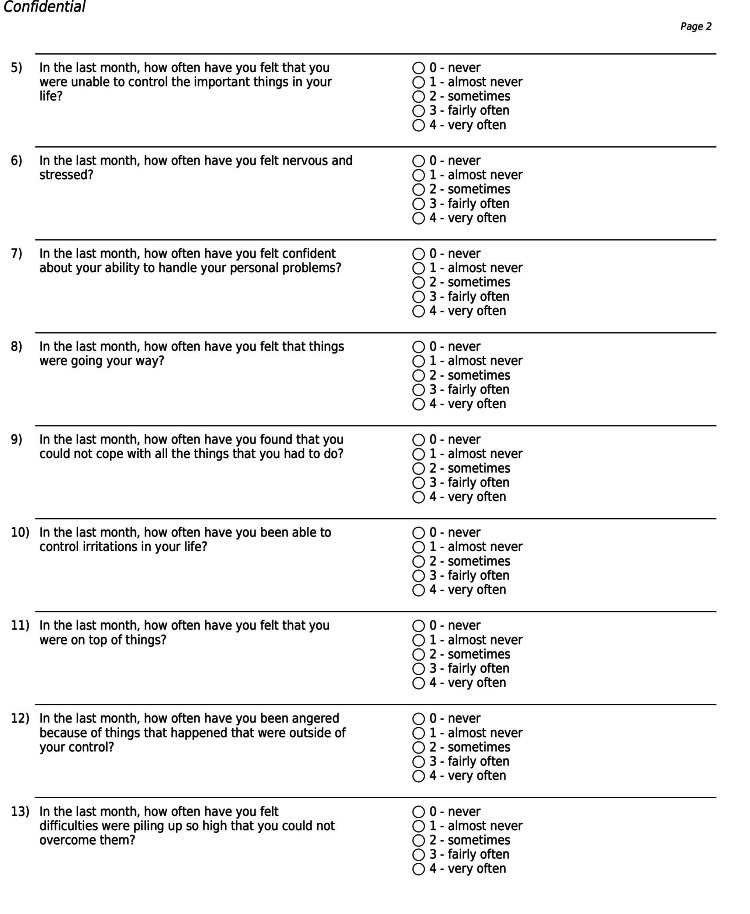
Pre-survey (page 2).

**Figure 3:**
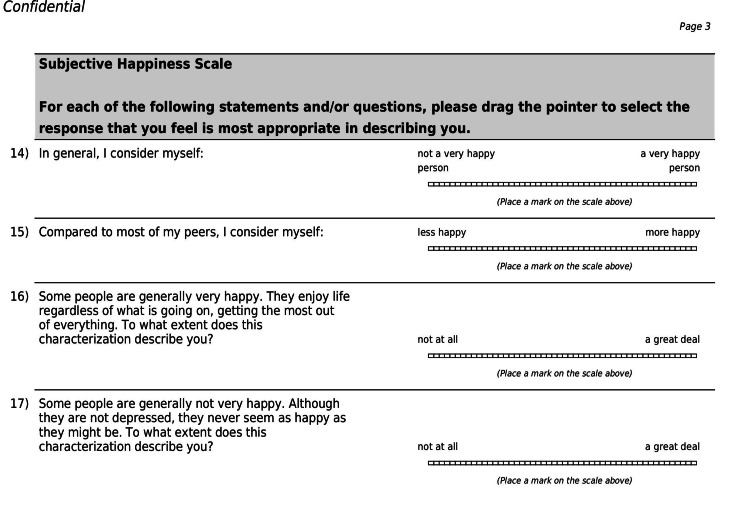
Pre-survey (page 3).

**Figure 4:**
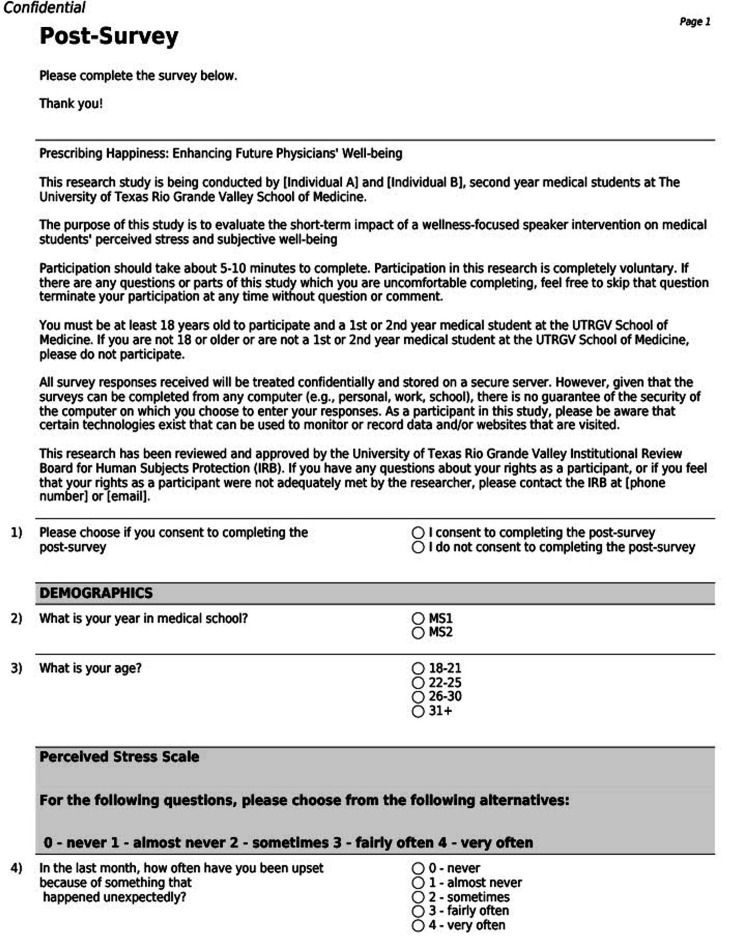
Post-survey (page 1).

**Figure 5:**
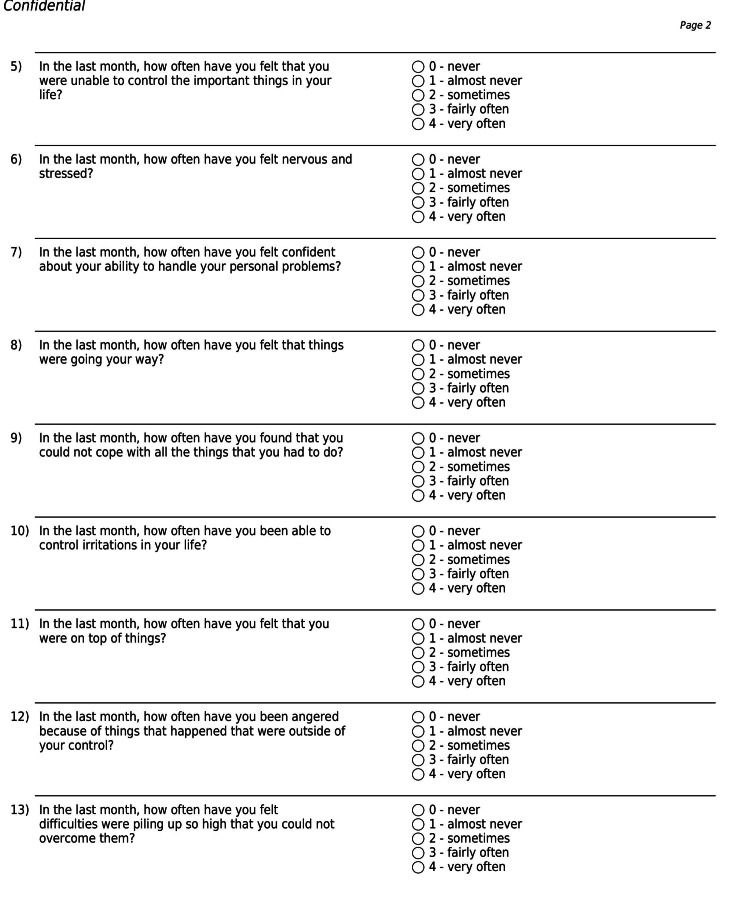
Post-survey (page 2).

**Figure 6:**
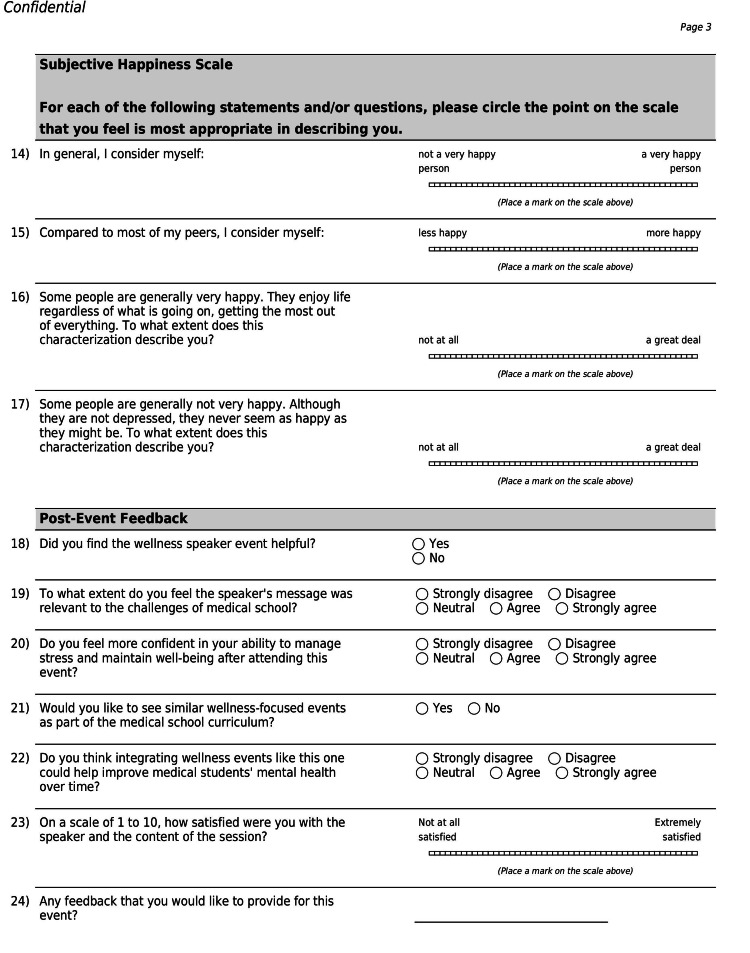
Post-survey (page 3).
